# “Opportunities and responsibilities”: how do pharmacists assess their professionalism?

**DOI:** 10.1186/s12909-024-05767-7

**Published:** 2024-08-01

**Authors:** Fernando de Castro Araújo-Neto, Aline Santana Dosea, Thaís Maria Araújo Tavares, Douglas de Menezes Santos, Alessandra Rezende Mesquita, Dyego Carlos Souza Anacleto de Araújo, Divaldo Pereira de Lyra-Jr

**Affiliations:** 1 Health Sciences Graduate Program, Laboratory of Teaching and Research in Social Pharmacy (LEPFS), Federal University of Sergipe, São Cristóvão, Sergipe, Brazil; 2 Graduate Program in Pharmaceutical Sciences, Laboratory of Teaching and Research in Social Pharmacy (LEPFS), Federal University of Sergipe, São Cristóvão, Sergipe, Brazil; 3 Laboratory of Teaching and Research in Social Pharmacy (LEPFS), Federal University of Sergipe, São Cristóvão, Sergipe, Brazil; 4 Department of Pharmaceutical Sciences, Laboratory of Innovation in Pharmaceutical Care, Federal University of Espírito Santo - Maruípe Campus, Vitória, Espírito Santo Brazil; 5 Department of Pharmacy, Laboratory of Teaching and Research in Social Pharmacy (LEPFS), Federal University of Sergipe, Cidade Universitária “Prof. José Aloísio Campos”, Jardim Rosa Elze, São Cristóvão, Sergipe, CEP: 49100-000 Brazil

**Keywords:** Pharmacy, Pharmacists, Professionalism, Assessment, Autonomy, Vocation, Professional council, Self-regulation, Continuing education, Altruism

## Abstract

**Introduction:**

Professionalism is fundamental to the existence of professions. In pharmacy, interest in this theme improved with events that examined the resocialization of pharmacists in care. With this, evaluating professionalism can help the operationalization of the theme and, consequently, the development of strategies for pharmacy consolidation before its challenges. Therefore, this study aimed to evaluate the professionalism of Brazilian pharmacists.

**Methods:**

To meet the objective, a cross-sectional study was conducted between March 2022 and August 2023. Data were collected using the Brazilian version of the “Modification of Hall’s Professionalism Scale for Use with Pharmacists”. The scale has 39 items grouped into the domains: autonomy, vocation, professional council, self-regulation, continuing education, and altruism. Data were analyzed using descriptive statistics and an ANOVA analysis of variance with post-hoc Hochberg or Games-Howell tests with Bootstrapping was conducted to verify differences between groups.

**Results:**

600 pharmacists participated in this study. The majority (69%) was female and carried out their professional activities in community pharmacies (50%). Professionalism scores ranged between 14 and 29 points, with an average of 22.8 points. Pharmacists working in outpatient clinics had higher scores in most factors, namely, altruism, continuing education, professional council, vocation, and autonomy. This indicates that the inclination of pharmacists to occupy areas focused on care can be significant to assess professionalism.

**Conclusions:**

The data obtained indicate that pharmacists working in outpatient clinics had higher professionalism scores compared to others. This corroborates the worldwide trend experienced by pharmacy in recent decades, which is the execution of increasingly patient-centered practice models.

## Introduction

Professionalism it is the guiding element between pharmacists and patients relationships reflected in behaviors and attitudes [1–4]. In the meantime, values historically associated with professionalism, such as autonomy and altruism, have been constantly encouraged in professionals, reinforcing appeals debated in medicine and dentistry, for example [2, 5]. In pharmacy, this is an old concern associated with recurrent questions about the suitability of the pharmaceutical work process before ethics in commercialization of products to detriment of service provision [6–9]. Effectively, this has provoked the profession to re-dimension its interests and objectives towards patients and health systems [10, 11].

To fulfill this purpose, it is necessary for pharmacists to understand the “opportunities and responsibilities” facing the dilemmas that are imposed on them, emphasizing the medicalization of society and the commodification of health. In addition, other phenomena that provoke traditional values of pharmacy and must be observed by professionals is the behavior and attitudes [10–13]. In this context, assessing professionalism is fundamental to understanding how the pharmacy has operationalized this topic, which is both important and challenging [4, 9, 14–16].

In countries such as Brazil, advances in consolidating professionalism associated with patient-centered work processes are timid compared to European countries. Thus, this assessment may be a response to the fear of invisibility due to delay, which is discussed in the literature [1, 14, 17–20]. Although cultural and demographic differences are barriers to a consensus on this theme, their assessment justifies global efforts to understand and disseminate professional behaviors among pharmacists [10, 20–24]. Therefore, the aim of this study was to assess the professionalism of Brazilian pharmacists.

## Method

### Study design and instrument

This cross-sectional study was carried out between March 2022 and August 2023, and it was evaluated the professionalism of pharmacists in Brazil. For data collection, the translated and adapted version of the “Modification of Hall’s Professionalism Scale for Use with Pharmacists” to Brazilian Portuguese language was used [25]. The report of this study followed the recommendations of “Strengthening the Reporting of Observational Studies in Epidemiology” (STROBE) [26].

This instrument was originally developed by Hall in 1968. Then adapted for pharmacists by Schack and Hepler in 1979. Finally, translated and adapted into Brazilian Portuguese by Araújo-Neto and collaborators. In this version, a cross-cultural adaptation was conducted, followed by a verification of content-based validity evidence using the Delphi technique [27, 28]. In this technique, geographically distinct experts gave their opinion on the content (questions) of the instrument. After this, the instrument was subjected to examination of its psychometric properties through exploratory factor analysis. This generated the final version of the instrument with 39 items and six factors. The score per item is a minimum of one point and a maximum of five points. The instrument’s total score is a minimum of six points and a maximum of 30 points [25, 29, 30].

 Tables1 and 2 presents the questions, factors, and their respective definitions.

**Table 1:**
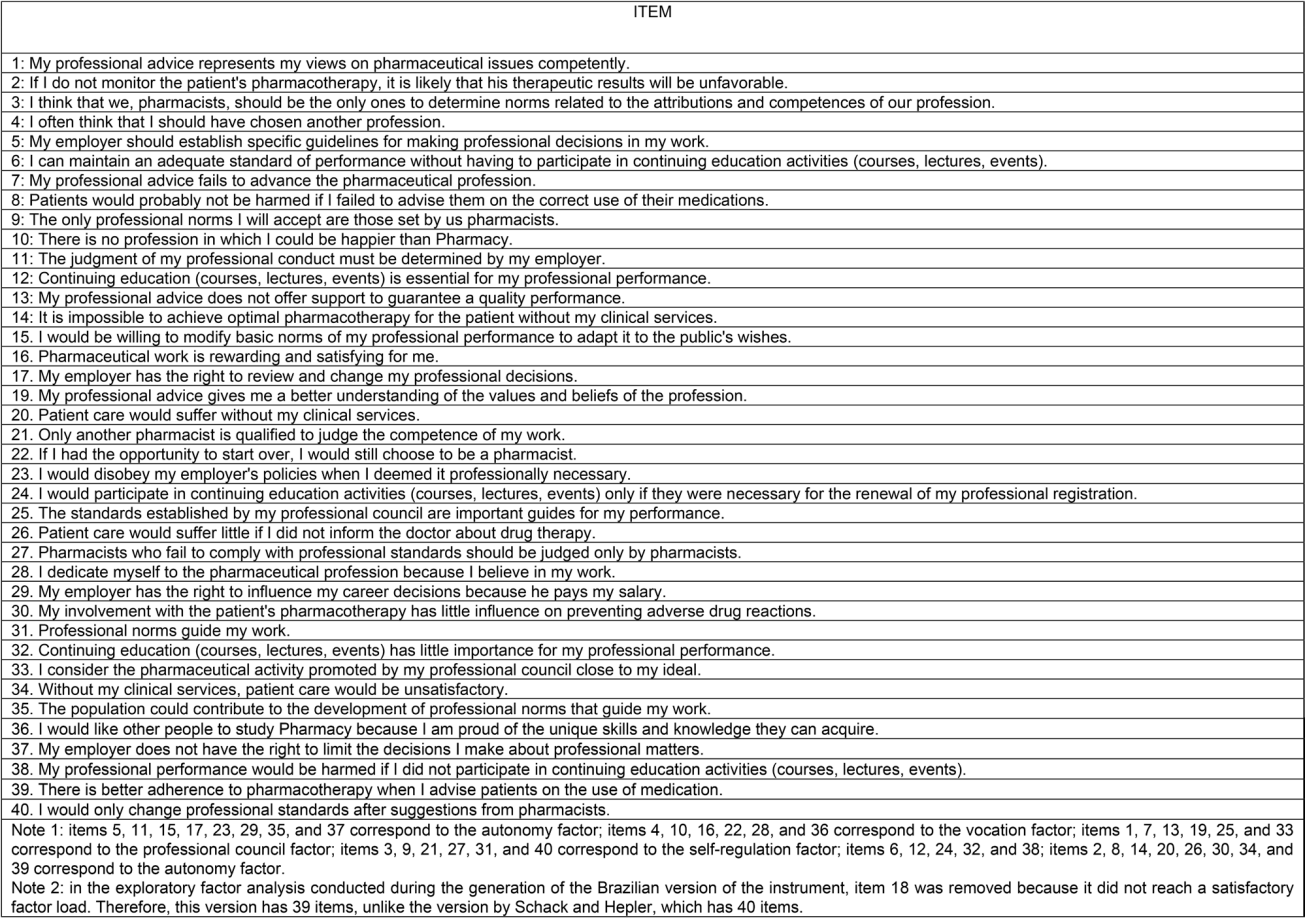
Brazilian version of the Modification of Hall’s Professionalism Scale for Use with Pharmacists

**Tablet 2:**
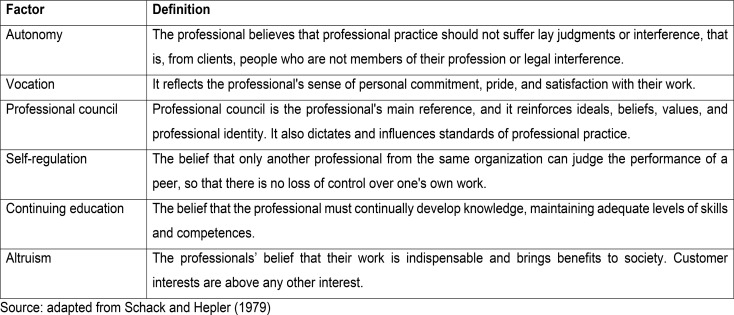
Professionalism factors adopted in the construction of the scale

### Study sample

Pharmacists from all geographic regions of Brazil who worked in the segments of community pharmacy, hospital pharmacy, public pharmacy, and pharmaceutical outpatient clinic were included in this study. According to official data, these occupational areas aggregated most of the pharmaceutical workforce in Brazil [31]. Furthermore, they are the areas of interest in the original version of the scale, developed by Schack and Hepler (1979).

### Sample size calculation

When considering the population of 234,301 pharmacists registered with the Federal Pharmacy Council of Brazil with a confidence level of 95% and a sampling error of 5%, it was considered that at least 384 pharmacists should participate in the study. To calculate this sample, the following formula was used: n = E2⋅(*N* − 1) + Z2⋅p⋅(1 − p)N⋅Z2⋅p⋅(1 − p).

### Data collection

Data collection took place using a virtual form, available on the Google forms platform, in which the participants accessed its content, the instructions for filling it out and, the informed consent form. The form was disseminated through the Federal Council of Pharmacy, the Laboratory of Teaching and Research in Social Pharmacy, as well as organizations, unions, and associations of Pharmacy practice social medias in Brazil.

The survey questionnaire included the following variables: gender (male or female), age, geographic region in which they reside (Midwest, Northeast, North, Southeast, South of Brazil), year of graduation in pharmacy, educational institution where studied pharmacy, (public, private, mixed or philanthropic), schooling (graduation, postgraduation (specialization), residency, master, and doctorate), type of institution of work (public, private, mixed, philanthropic, or self-employed), and occupational area in which they work professionally (community pharmacy, hospital pharmacy, public pharmacy, or pharmaceutical outpatient clinic).

Philanthropic institutions are associations that provide services on a non-profit basis; mixed institutions mix resources from public and private initiatives in their financial capital. The public pharmacy is a place where medicines are dispensed under Brazilian National Public Healthcare System (SUS). They are generally, part of Primary Health Care. According to Brazil’s Federal Pharmacy Council (2022), pharmaceutical outpatient clinics are places that operate autonomously or are linked to other commercial establishments and are intended for private patient care [32–35].

### Data analysis

Data were charted using Microsoft Excel. The characteristics of participants were presented using descriptive statistics (absolute and relative frequency, mean, and standard deviation).

Student’s t test for independent samples was used to investigate differences in professionalism scores according to gender. Analysis of variance (ANOVA – One Way) were performed to verify differences in professionalism scores according to education, area in which they work professionally (occupational area), and type of institution where they develop professional activities. The homogeneity of variance was evaluated using Levene’s test. The Hochberg test was used when the assumption of equal variances was met, while the Games-Howell test was used when the assumption was violated. The bootstrapping procedure (1000 re-samplings; 95% CI BCa) was adopted to correct possible deviations from normality and differences between group sizes, in addition to presenting a 95% confidence interval for differences between means [36, 37].

### Ethical considerations

This study was developed in accordance with the principles established in the resolution nº 466/12 of the National Health Council of Brazil, being approved by the Ethics Committee in Research with Humans of the Federal University of Sergipe, under protocol number 4,169,752.

## Results

### Characteristics of the participants

A total of 600 pharmacists participated in the study and the majority (*N* = 414; 69%) was female. The average age of participants was 34 years. Most pharmacists develop professional activities in private institutions (*N* = 335; 55.85%) and in community pharmacies (*N* = 298; 50%). Table 3 gathers the data regarding the characterization of participants.

**Table 3:** Characterization of study participants (*n* = 600)

Characteristics		*N* (%)
Sex	Male	189 (31%)
	Female	414 (69%)
Age (mean and standard deviation)		34 (8.36)
Year of graduation in pharmacy	2020–2022	149 (25%)
	2010–2019	317 (53%)
	2000–2009	92 (15%)
	1990–1999	35 (5.84%)
	1980–1989	6 (1%)
	1970–1979	1 (0.16%)
Educational institution where studied pharmacy	Private	351 (58.5%)
	Public	249 (41.5%)
Schooling	Graduation	181 (30.2%)
	Postgraduation (Specialization)	277 (46.2%)
	Residency	44 (7.3%)
	Master	75 (12.5%)
	Doctorate	23 (3.8%)
Region of residence	Midwest	36 (6%)
	Northeast	228 (38%)
	North	56 (9.5%)
	Southeast	179 (29.7%)
	South	101 (16.8%)
Type of institution of work	Self-employed	21 (3.5%)
	Philanthropic	14 (2.3%)
	Mixed	48 (8%)
	Private	335 (55.85%)
	Public	182 (30.35%)
Occupational area	Pharmaceutical outpatient clinic	31 (5.2%)
	Community Pharmacy	298 (50%)
	Hospital Pharmacy	170 (28%)
	Public Pharmacy	101 (16.8%)

### Evaluation of professionalism among Brazilian pharmacists

The total professionalism scores among pharmacists ranged from 14 to 29 points, with an average of 22.8 points, as shown in Table 4.

**Table 4:** Mean of total score and factorial score of the Brazilian version of the “Modification of Hall’s professionalism scale for Use with Pharmacist”

	Minimum	Maximum	Mean (SD)	IC 95%
Total score	14	29	22.8 (2.3)	22.6–23
Altruism	2	5	4.2 (0.6)	4.1–4.2
Continuing education	1	5	4.5 (0.6)	4.4–4.5
Self-regulation	2	5	3.4 (0.6)	3.3-0.34
Professional Council	1	5	3.3 (0.9)	3.2–3.4
Vocation	1	5	3.6 (1.0)	3.5–3.7
Autonomy	2	5	3.8 (0.6)	3.8–3.9

The comparison between the total professionalism scores and the Brazilian-version scale factors shown in Table 5, presents values within the mean, with emphasis on the means of pharmacists working in outpatient clinics (mean 23.9; standard deviation 2.1). A simple difference was observed between means by sex, with female professionals having higher scores (mean 22.8; standard deviation 2.3), however, this was not considered a statistically significant difference (*p* = 0.45).

**Table 5:** Comparison between total scores and factorial scores of the Brazilian version of the scale

	Total score		Altruism		Continuing education		Self-regulation		Professional council		Vocation		Autonomy	
	Mean (SD)	*p*	Mean (SD)	*p*	Mean (SD)	*p*	Mean (SD)	*p*	Mean (SD)	*p*	Mean (SD)	*p*	Mean (SD)	*p*
**Sex**														
Male	22.6 (2.4)	0.45	4.2 (0.60)	0.59	4.4 (0.62)	**0.01**	3.50 (0.65)	**0.001**	3.30 (0.91)	0.87	3.52 (0.96)	0.14	3.77 (0.61)	0.51
Female	22.8 (2.3)		4.2 (0.50)		4.5 (0.57)		3.31 (0.65)		3.32 (0.95)		3.65 (0.95)		3.81 (0.55)	
**Type of institution of work**														
Self-employed	22.5 (2.77)	0.58	4.4 (0.45)	0.28	4.3 (0.96)	0.34	3.4 (0.79)	**< 0.001**	3.2 (1.0)	0.84	3.4 (0.86)	0.92	3.8 (0.53)	0.99
Philanthropic	23.2 (1.99)		4.4 (0.58)		4.6 (0.38)		3.3 (0.52)		3.4 (0.80)		3.7 (0.77)		3.8 (0.66)	
Mixed	22.8 (2.34)		4.2 (0.54)		4.5 (0.53)		3.4 (0.71)		3.2 (0.88)		3.6 (0.97)		3.8 (0.60)	
Private	22.9 (2.36)		4.2 (0.56)		4.5 (0.58)		3.5 (0.62)		3.3 (0.94)		3.6 (0.99)		3.8 (0.58)	
Public	22.5 (2.22)		4.1 (0.59)		4.6 (0.57)		3.1 (0.65)		3.3 (0.95)		3.6 (0.90)		3.8 (0.60)	
**Schooling**														
Graduation	22.8 (2.36)	0.08	4.2 (0.56)	0.38	4.3 (0.70)	**< 001**	3.5 (0.58)	**< 0.001**	3.4 (0.88)	0.32	3.7 (0.96)	**0.03**	3.8 (0.60)	**< 0.001**
Specialization	22.8 (2.31)		4.2 (0.55)		4.6 (0.50)		3.4 (0.66)		3.3 (0.96)		3.6 (0.96)		3.8 (0.60)	
Residency	22.0 (2.19)		4.1 (0.72)		4.5 (0.73)		3.2 (0.66)		3.1 (0.85)		3.3 (0.93)		3.8 (0.48)	
Master	23.1 (2.21)		4.3 (0.53)		4.7 (0.41)		3.2 (0.70)		3.2 (0.97)		3.8 (0.84)		3.9 (0.55)	
Doctorate	22.2 (2.55)		4.1 (0.60)		4.6 (0.49)		2.9 (0.64)		3.3 (0.98)		3.5 (1.04)		3.9 (0.59)	
**Educational institution where studied pharmacy**														
Public	22.5 (2.22)	0.14	4.1 (0.60)	0.34	4.6 (0.57)	0.12	3.1 (0.65)	**< 0.001**	3.3 (0.95)	0.84	3.6 (0.90)	0.93	3.8 (0.60)	0.87
Private/Mixed/ Philanthropic	22.8 (2.36)		4.2 (0.56)		4.5 (0.60)		3.5 (0.64)		3.3 (0.92)		3.6 (0.98)		3.8 (0.58)	
**Occupational area**														
Pharmaceutical outpatient clinic	23.9 (2.1)	**< 001**	4.4 (0.34)	**0.006**	4.7 (0.51)	**0.007**	3.2 (0.68)	**< 0.001**	3.6 (0.94)	**0.03**	3.9 (0.89)	**0.26**	4.0 (0.48)	**0.001**
Community Pharmacy	22.9 (2.4)		4.2 (0.57)		4.4 (0.63)		3.5 (0.61)		3.4 (0.94)		3.6 (1.0)		3.8 (0.59)	
Hospital Pharmacy	22.2 (2.3)		4.1 (0.59)		4.5 (0.56)		3.2 (0.66)		3.2 (0.89)		3.5 (0.90)		3.7 (0.64)	
Public Pharmacy	22.9 (2.1)		4.1 (0.57)		4.6 (0.51)		3.2 (0.65)		3.4 (0.97)		3.6 (0.88)		3.9 (0.49)	

Scores by scale factor (altruism, continuing education, self-regulation, professional council, vocation, autonomy) and variables were also compared. In this context, pharmacists working in outpatient clinics had higher scores than professionals from other occupational areas for most factors, namely, altruism, continuing education, professional council, vocation, and autonomy.

Comparisons between groups performed in ANOVA using post-hoc tests showed significant differences, with emphasis on the total score of pharmacists who carry out professional activities in outpatient clinics compared to hospital pharmacists or those working in public pharmacies. Table 6 presents data referring to the post-hoc Test with Bootstrapping (95% CI Bca) for the variables that showed a statistically significant difference in the ANOVA of the “Modification of Hall’s Professionalism Scale for Use with Pharmacists” Brazilian version.

**Table 6:** Bootstrapping post-hoc test for variables that showed statistically significant difference in Anova of Scale

	Variable	Comparison between groups		Difference between means	*Bootstrapping*		
					Standard model	Confidence Interval BCa 95%	
						Bottom	Upper
Total score	Occupational area *Hochberg’s Bootstrapping post-hoc test (95% IC Bca)*	Pharmaceutical outpatient clinic	**Community Pharmacy**	**0.965**	**0.427**	**0.106**	**1.867**
			**Hospital Pharmacy**	**1.676**	**0.441**	**0.748**	**2.601**
			**Public Pharmacy**	**1.025**	**0.447**	**0.178**	**1.83**
		Community Pharmacy	**Hospital Pharmacy**	**0.711**	**0.216**	**0.281**	**1.102**
			Public Pharmacy	0.06	0.239	-0.366	0.433
		Hospital Pharmacy	**Public Pharmacy**	**-0.651**	**0.267**	**-1.144**	**-0.192**
**Altruism**	Occupational area *Hochberg’s Bootstrapping post-hoc test (95% IC Bca)*	Pharmaceutical outpatient clinic	**Community Pharmacy**	**0.215**	**0.069**	**0.066**	**0.356**
			**Hospital Pharmacy**	**0.258**	**0.075**	**0.114**	**0.404**
			**Public Pharmacy**	**0.258**	**0.084**	**0.071**	**0.432**
		Community Pharmacy	Hospital Pharmacy	0.043	0.054	-0.062	0.154
			Public Pharmacy	0.043	0.067	-0.083	0.164
		Hospital Pharmacy	Public Pharmacy	0	0.072	-0.146	0.141
**Continuing Education**	Schooling *Games-Howell’s Bootstrapping post-hoc test* *(95% IC Bca)*	Graduation	**Specialization**	**-0.261**	**0.06**	**-0.382**	**-0.145**
			Residency	-0.211	0.124	-0.429	0.053
			**Master**	**-0.367**	**0.066**	**-0.492**	**-0.233**
			**Doctorate**	**-0.254**	**0.111**	**-0.469**	**-0.031**
		Specialization	Residency	0.05	0.117	-0.149	0.292
			Master	-0.105	0.055	-0.226	0.007
			Doctorate	0.007	0.102	-0.195	0.214
		Residency	Master	-0.156	0.121	-0.419	0.06
			Doctorate	-0.043	0.146	-0.327	0.232
		Master	Doctorate	0.113	0.108	-0.104	0.322
	Occupational area *Hochberg’s Bootstrapping post-hoc test (95% IC Bca)*	Pharmaceutical outpatient clinic	**Community Pharmacy**	**0.303**	**0.1**	**0.064**	**0.492**
			Hospital Pharmacy	0.217	0.103	-0.013	0.415
			Public Pharmacy	0.135	0.109	-0.121	0.353
		Community Pharmacy	Hospital Pharmacy	-0.086	0.055	-0.193	0.027
			**Public Pharmacy**	**-0.168**	**0.062**	**-0.295**	**-0.027**
		Hospital Pharmacy	Public Pharmacy	-0.082	0.068	-0.216	0.06
**Self-regulation**	Type of institution of work *Hochberg’s Bootstrapping post-hoc test (95% IC Bca)*	Self-employed	Philanthropic	0.135	0.228	-0.295	0.569
			Mixed	0.063	0.206	-0.325	0.451
			Private	-0.037	0.184	-0.35	0.314
			Public	0.301	0.184	-0.03	0.67
		Philanthropic	Mixed	-0.072	0.168	-0.429	0.245
			Private	-0.172	0.136	-0.454	0.11
			Public	0.166	0.141	-0.127	0.429
		Mixed	Private	-0.1	0.108	-0.326	0.117
			**Public**	**0.238**	**0.115**	**0.002**	**0.457**
		Private	**Public**	**0.338**	**0.058**	**0.224**	**0.445**
	Schooling *Games-Howell’s Bootstrapping post-hoc test* *(95% IC Bca)*	Graduation	**Specialization**	**0.163**	**0.057**	**0.041**	**0.271**
			**Residency**	**0.345**	**0.107**	**0.119**	**0.582**
			**Master**	**0.282**	**0.09**	**0.13**	**0.45**
			**Doctorate**	**0.657**	**0.143**	**0.384**	**0.902**
		Specialization	Residency	0.182	0.107	-0.033	0.431
			Master	0.12	0.091	-0.053	0.289
			**Doctorate**	**0.494**	**0.139**	**0.216**	**0.763**
		Residency	Master	-0.063	0.128	-0.305	0.172
			Doctorate	0.312	0.169	-0.011	0.59
		Master	**Doctorate**	**0.375**	**0.154**	**0.078**	**0.652**
	Occupational area *Games-Howell’s Bootstrapping post-hoc test* *(95% IC Bca)*	Pharmaceutical outpatient clinic	Community Pharmacy	-0.301	0.126	-0.548	-0.036
			Hospital Pharmacy	0.057	0.13	-0.208	0.328
			Public Pharmacy	0.055	0.137	-0.211	0.332
		Community Pharmacy	Pharmaceutical outpatient clinic	0.301	0.126	0.036	0.548
			Hospital Pharmacy	0.357	0.061	0.239	0.476
			Public Pharmacy	0.356	0.074	0.214	0.499
		Hospital Pharmacy	Pharmaceutical outpatient clinic	-0.057	0.13	-0.328	0.208
			Community Pharmacy	-0.357	0.061	-0.476	-0.239
			Public Pharmacy	-0.001	0.079	-0.154	0.15
		Public Pharmacy	Pharmaceutical outpatient clinic	-0.055	0.137	-0.332	0.211
			Community Pharmacy	-0.356	0.074	-0.499	-0.214
			Hospital Pharmacy	0.001	0.079	-0.15	0.154
**Professional Council**	Occupational area *Hochberg’s Bootstrapping post-hoc test (95% IC Bca)*	Pharmaceutical outpatient clinic	Community Pharmacy	0.25	0.174	-0.098	0.592
			**Hospital Pharmacy**	**0.45**	**0.176**	**0.1**	**0.802**
			Public Pharmacy	0.228	0.196	-0.19	0.635
		Community Pharmacy	**Hospital Pharmacy**	**0.2**	**0.086**	**0.038**	**0.361**
			Public Pharmacy	-0.023	0.109	-0.235	0.191
		Hospital Pharmacy	Public Pharmacy	-0.223	0.118	-0.455	0.033
**Vocation**	Schooling *Games-Howell’s Bootstrapping post-hoc test* *(95% IC Bca)*	Graduation	Specialization	0.137	0.089	-0.023	0.315
			**Residency**	**0.355**	**0.151**	**0.056**	**0.641**
			Master	-0.139	0.12	-0.375	0.108
			Doctorate	0.21	0.229	-0.211	0.661
		Specialization	Residency	0.218	0.146	-0.076	0.512
			Master	-0.276	0.115	-0.514	-0.028
			Doctorate	0.073	0.228	-0.368	0.521
		Residency	Master	-0.493	0.172	-0.833	-0.125
			Doctorate	-0.145	0.263	-0.673	0.421
		Master	Doctorate	0.348	0.245	-0.125	0.789
**Autonomy**	Schooling *Games-Howell’s Bootstrapping post-hoc test* *(95% IC Bca)*	Graduation	Specialization	-0.061	0.058	-0.173	0.047
			Residency	-0.036	0.087	-0.209	0.128
			Master	-0.101	0.082	-0.261	0.043
			Doctorate	-0.135	0.133	-0.381	0.135
		Specialization	Residency	0.025	0.082	-0.136	0.183
			Master	-0.04	0.075	-0.189	0.108
			Doctorate	-0.074	0.131	-0.329	0.197
		Residency	Master	-0.065	0.097	-0.243	0.125
			Doctorate	-0.099	0.147	-0.387	0.22
		Master	Doctorate	-0.034	0.14	-0.296	0.224
	Occupational area *Hochberg’s Bootstrapping post-hoc test (95% IC Bca)*	Pharmaceutical outpatient clinic	**Community Pharmacy**	**0.21**	**0.089**	**0.012**	**0.389**
			**Hospital Pharmacy**	**0.33**	**0.095**	**0.139**	**0.526**
			Public Pharmacy	0.09	0.096	-0.11	0.285
		Community Pharmacy	**Hospital Pharmacy**	**0.12**	**0.059**	**0.012**	**0.238**
			Public Pharmacy	-0.12	0.06	-0.242	0.004
		Hospital Pharmacy	Public Pharmacy	-0.24	0.07	-0.367	-0.112

In altruism, scores comparing pharmacists who work in outpatient clinics and in other places of activity stood out. As for continuing education, there were significant findings in the differences between pharmacists with postgraduation degree, such as specialization, master’s, and doctorate, compared to professionals without postgraduation.

In the self-regulation factor, the higher scores stood out among pharmacists who develop their professional activities in the private sector when compared with professionals from public institutions, as well as in the comparison between pharmacists that do not have a postgraduation degree. Still regarding this factor, in comparisons by occupational areas, the averages for community pharmacy were higher than those for the other groups.

As for the professional council factor, the comparisons about the place where pharmacists carry out professional activities showed significant highlight for the community pharmacy compared to the hospital pharmacy. Regarding the vocation factor, the results of the Games-Howell post-hoc test identified significant differences in the means of pharmacists who do not have a postgraduation degree, compared to resident pharmacists. For the autonomy factor, the Hochberg post-hoc test observed differences in the average of pharmacists who work in outpatient clinics, compared to community and hospital pharmacists. Likewise, the averages of community pharmacists were higher compared to hospital pharmacists, as well as among pharmacists whose maximum level of education was a master’s or doctorate.

## Discussion

In this study, the professionalism of Brazilian pharmacists was assessed using the Brazilian-Portuguese version of the “Modification of Hall’s Professionalism Scale for Use with Pharmacists” [25]. Regarding the scale total score, significant findings were observed in the evaluation of pharmacists who work in outpatient clinics. In Brazil, outpatient clinics are places where pharmacists provide private care to their patients, and it may be linked to other health establishments or be independent [35].

The literature considers interactions between pharmacists and patients as the “heart of pharmacy”. A “fiducial” relationship with the patient is seen as the moral compass of profession, which has been encouraged in recent years especially through the transformation of professionalism into a construct that can be evaluated through behaviors and attitudes performed by pharmacists [21, 38–40]. Currently, pharmacy is interested in this phenomenon, which is common in professions such as medicine and nursing, as these events are opportunities for pharmacists to resocialize to patient care as a model of professional practice [14, 21, 39, 41, 42].

### Altruism

Altruism, considered by the literature as a central value in the practice of health professionals, was also significantly observed in the scores of pharmacists who work in private outpatient clinic [43, 44]. In addition to theoretical models of professionalism endorsing the importance of professionals’ altruistic attitudes towards patients, it is possible to infer that places such as outpatient clinic seem to be preferable to the development of more private relationships with the patient, as opposed to scenarios such as community pharmacy [3, 45, 46].

In those places, the possibility of building a relationship of trust between pharmacists and their patient is possible, due to the nature of the structure and processes present there, as well as the possibility of performing more complex clinical services, for example, pharmacotherapy review [9, 47]. This does not mean that altruistic attitudes are restricted to such environments. Essentially, there are potential challenges and ethical conflicts anywhere, therefore pharmacists are expected to resize their skills in order to meet the needs of patients, especially since these places are the most accessible health establishment for users of health services [48].

### Continuing education

Esoteric and non-transferable knowledge is one of the essential characteristics in the separation between professionals and amateurs [49–51]. Understanding that professional learning is not restricted to academic training, the literature has highlighted the need to provide continuing education to pharmacists, due to constant social demands for qualified professionals to carry out their services [52, 53]. Similar observations are identified in studies developed among physicians and nurses that reported the success of educational programs for patient safety [52, 54–56].

In the present study, pharmacists with postgraduation degree, such as specialization, master’s, and doctorate, were better evaluated regarding their perceptions on this theme, compared to professionals that only graduated. According to the literature, there is no curricular standardization in the pharmacy teaching and each country organizes its guidelines according to local demands and the regulation of professional attributions [57]. Thus, despite pharmaceutical care is seen as strategic for the occupational survival of pharmacy, its integral teaching seems to be a distant reality in countries such as Brazil, Germany, Jordan, and Saudi Arabia [17, 18, 57–61].

In this context, as the roles of pharmacists migrate to meet clinical demands, the skills necessary to achieve this competence must be taught and, above all, endorsed during their professional careers, through continuing education actions [57]. Thus, the adoption of these programs by pharmacists and their employers can expand the scope of clinical activities, to detriment of tasks that recruit less cognitive effort, and help to maximize the influence of pharmacists in clinical decision making [62–64].

### Self-regulation

In the literature, self-regulation is a construct commonly associated with the ability of a profession to determine guidelines for its own professional practice, based on exclusive skills acquired in academic training [49–51, 59, 65, 66]. The functionalist perspective of the study of professions has considered that professionals as pharmacists are not able to determine the dimensions of their work processes, contradicting the discourse of professional sovereignty and the influence of organizations that regulate the exercise of these professions [59, 67].

In Brazil, most community pharmacists carry out their professional activities in pharmacies linked to medicine retail. That is, private, for-profit pharmacies [17, 18, 31, 59]. Even though Brazilian legislation guarantees that pharmacists are the highest authority in these environments and that pharmacy owners must comply with the technical decisions of these professionals, the reality is contradictory [59, 67, 68]. The experiences of Canada and Ireland, for example, point to difficulties in controlling their own work, in addition to the multiple roles performed in exceptional situations, such as the COVID-19 pandemic [69, 70].

Despite these challenges, it is possible to assume that the belief of control over one’s work in this environment is related to professional identity, with the feeling of responsibility that pharmacists have towards their patients, as they are generally the only healthcare professional available there and obviously, due to legislation [3, 68, 71]. As guidance to address this crisis, it is incumbent upon organizations and professional councils to possess the capability to influence and ensure the integration of necessary attributes into pharmacy practice. This integration is essential to meet social demands and, consequently, ensure the sustainability of the pharmacy profession [67, 72].

### Professional council

According to the literature, professional councils are the maximum representation of a profession in a country, being responsible for regulating the exercise of professional practice, inspection, surveillance, and punishment of professionals who transgress the deontological norms that are established by the councils themselves [17, 18, 59, 73]. This model of regulation through a professional association or organization is widely debated in the literature and seen by the sociology of professions as a unique criterion for benefits such as self-regulation and professional autonomy [50, 74, 75].

Interestingly, in this study, the scores for this factor showed differences between professionals from different occupational areas, with significant emphasis on community pharmacists. Today, in Brazil, despite the administrative governance of the Federal Council of Pharmacy contemplates advances in all 135 occupational areas regulated for professional practice of pharmacists, there is a discreet situation that can justify this phenomenon [18, 31, 59].

Regulatory devices such as the Federal Law 5991/1973 did not even name the pharmacist as the professional responsible for the clinical management of community pharmacies. Conversely, the term “technical responsible” contained in the law, opened up margins for lay people to claim responsibility for these establishments, sidelining pharmacists for duties far from the clinic [18, 73, 76, 77]. From the 2000s onwards community pharmacy did return to the spotlight through events that culminated in the creation of a National Policy for Access to Essential Medicines, the regulation of clinical duties of pharmacists and the creation of the Federal Law 13,021/2014 which treats these places as health facilities [18, 77, 78]. Thus, being an auxiliary mechanism to the health system, the mandatory presence of pharmacists was guaranteed, and it was elevated to the position of service provider, to the detriment of the past of an unethical salesperson.

In this sense, community pharmacists who, in Brazil, make up the majority of the profession’s workforce and are distributed among more than 114,00 establishments, have become the greatest pharmacy showcase, in Brazil and the world, and a major focus of actions by their professional organization. To the literature, this may be part of the profession’s strategic effort in search of visibility, prestige, and power [18, 31, 49, 50, 79]. The approach to the health system and patients through the provision of services according to social needs is, historically, the main mark of a profession and this is observed as more responsibilities are entrusted to these professionals.

Another aspect is the organizational model of community pharmacies. [59]. In hospitals, the division of work still positions the physician as a leader, which impacts on the decisions taken by other professionals, who routinely depend on medical approvals. For hospital pharmacists, this scenario may be an indicator that justifies the lower identification with their professional council, compared to professionals in “showcase” areas [80–87].

### Vocation

In the present study, pharmacists who were only graduated had higher scores in the vocation factor, compared to professionals who had a residency degree. In the functionalist literature, vocation is one of the fundamental elements in the formation of professional identity. This principle finds support in classic definitions of profession, which associate such terms with a biblical logic, however far from the current reality and the degree to which professionals are immersed in conflicts in the world of work [50, 85, 88, 89]. Despite this, from a bureaucratic and organizational point of view, it seems to be useful to encourage the vocational logic in choosing a profession and in the formation of the identity of individuals who are looking for an occupation.

With the disenchantment of the world and the naturalization of science as a model of responses to everyday phenomena, professions acquired a new status that moves away from the divine idea, however, it takes advantage of this by uttering through ideologies that they are the only alternatives facing the resolution of social demands [50, 74, 88–91]. In the meantime, the discourse of professional identity advances as it seems clear that professional socialization of individuals depends on their level of social identification with their profession. Therefore, the belief of belonging to an organization is still a fundamental part of professional development [92]. Although the residency is an interesting space for the development of professional skills, in these places there are possibilities for conflicts and contradictions that, certainly, can affect professional self-perception about their roles, identity, and consequently, feelings about their vocation [93, 94].

### Autonomy

Finally, the fundamental constituent of professions emerges as a factor discussed in this scale, as it compares whether pharmacists working in clinics tend to have higher scores than professionals in other occupational areas evaluated. Interestingly, the scores of community pharmacists overlapped those of hospital pharmacists. Professions have gone through movements that make them increasingly proletarianized, deregulated, and incapable of managing their own practice models. In the literature, the self-perception of pharmacists about external interference in their work has shown high rates of dissatisfaction with the profession [95]. In pharmacy, alienated work is observed in processes of occupational areas such as community pharmacy and hospital pharmacy [85, 96–98]. Attributions that could be delegated to professionals with a lower level of degree, salary policies that are incompatible with living costs, work processes that do not involve patient participation, business models that resemble “fast-food” logic and do not prioritize quality of services are among the criticisms [99–102]. Despite changes in the roles of pharmacists in these places, dissatisfaction with work ends up becoming part of everyday life [97, 103–106].

According to the findings of this study, it is possible to infer that outpatient clinics can be viable alternatives for pharmacists to control their own work. Although, in this model pharmacists receive patients referred by physicians, there is the possibility of developing more complex services such as, pharmacotherapy review, and pharmacotherapeutic follow-up, stepping back of dispensing, which although necessary, is still seen as fragile and incipient [8, 12, 39, 107–112]. Another highlight is the possibility of entrepreneurial practices in these places, giving pharmacists greater freedom to make choices and decisions about themselves, their services, and the relationships with patients [113, 114].

In the literature, entrepreneurial practices symbolize autonomy, deep relationships with patients and, release of physicians submission [115–118]. However, entrepreneurship should not be used as an alternative to the cooling of work relations. On the opposite, it should be encouraged with tax incentives and in work processes, but not as support for professionals who seek to migrate from employment relationships that are increasingly neglected by working conditions and worker well-being. Likewise, it would not be opportune for the occupational survival of pharmacy to completely abandon community pharmacies and hospitals with migration to this new business model, once these places are where the pharmaceutical workforce is required the most, as identified in this study [12, 59, 85, 108, 109, 111, 119–126]. Among the reasons that influence pharmacists to migrate to an academic career, for example, are salaries, the possibility of working towards the future of the profession and autonomy [127]. The lack of autonomy is one of the reasons for the decline in interest in pharmacy as a professional career [128]. In academic environments where the population with master’s or doctorate degrees is concentrated, the reality is discrepant from occupational areas such as community pharmacies and hospitals. For example, there are differences in salary policy, with higher salaries for those who wish to pursue an academic career. As already mentioned, the feeling of autonomy is also superior, in addition to professional satisfaction, workload and professional career prospects [127–130].

### Strengths and limitations

To our knowledge, this study presents an unprecedented report of Brazilian pharmacists’ assessment of professionalism, using a self-administered instrument and with evidence of content and construct validity [25]. Although the sample calculation indicates representativeness, other applications of the instrument are appropriate to minimize biases related to the geographic concentration of participants, which was high in the Northeast region and low in the North and Midwest regions. Furthermore, the number of items in the instrument, 39, may also have caused fatigue or abandonment on the part of some participants, which can be captured in future applications through instruments capable of identifying and providing data on such hypotheses. During data collection, it was also not possible to obtain a list of postgraduate courses taken by study participants. This variable will be well used in future studies that use this instrument as a data collection tool.

## Conclusion

In this study, the professionalism of Brazilian pharmacists was evaluated, with significant scores for professionals working in outpatient clinics. These findings show that despite the ambiguity still imposed on pharmacy work processes through dilemmas regarding professionalism, the profession has sought alternatives that combine political articulation, technical qualification, and behavioral development to improve its workforce before the challenges of the 21st century and post pandemic.

## Data Availability

The datasets used and/or analysed during the current study available from the corresponding author on reasonable request.

## Declarations

### Dual publication

The results in this manuscript have not been published elsewhere, nor are they under consideration by another publisher.
